# Bacterial community composition and potential pathogens along the Pinheiros River in the southeast of Brazil

**DOI:** 10.1038/s41598-020-66386-y

**Published:** 2020-06-09

**Authors:** Rafaela Garrido Godoy, Marta Angela Marcondes, Rodrigo Pessôa, Andrezza Nascimento, Jefferson Russo Victor, Alberto José da Silva Duarte, Patricia Bianca Clissa, Sabri Saeed Sanabani

**Affiliations:** 1 Laboratory of Dermatology and Immunodeficiency, São Paulo University Medical School, Sao Paulo, SP Brazil; 2 Department of Microbiology, Fedral University of São Caetano, São Paulo, Brazil; 3 Division of Environmental Health, Faculdades Metropolitanas Unidas (FMU), Laureate International Universities, São Paulo, Brazil; 4 Division of Pathology, Medical School, University of São Paulo, São Paulo, Brazil; 5 Immunopathology Laboratory, Butantan Institute, Sao Paulo, SP Brazil; 6 Laboratory of Medical Investigation LIM 03, Hospital das Clínicas (HCFMU), School of Medicine, University of São Paulo, São Paulo, Brazil

**Keywords:** Environmental impact, Microbial ecology

## Abstract

The Pinheiros River in São Paulo, Brazil, crosses through the capital city and has its confluence with the River Tiete, which comprises several reservoirs along its course. Although Pinheiros River is considered one of the heaviest polluted rivers in Brazil, little is known about its bacterial composition, their metabolic functions or how these communities are affected by the physicochemical parameters of the river. In this study, we used the 16S rRNA gene Illumina MiSeq sequencing to profile the bacterial community from the water surface at 11 points along the course of the River. Taxonomical composition revealed an abundance of *Proteobacteria* phyla, followed by *Firmicutes* and *Bacteroidetes*, with a total of 233 classified bacterial families and 558 known bacterial genera. Among the 35 potentially pathogenic bacteria identified, *Arcobacter* was the most predominant genus. The disrupted physicochemical parameters detected in this study may possibly contribute to the composition and distribution of the bacterial community in the Pinheiros River. Predictive functional analysis suggests the River is abundant in motility genes, including bacterial chemotaxis and flagellar assembly. These results provide novel and detailed insights into the bacterial communities and putative function of the surface water in the Pinheiros River.

## Introduction

Land-use and urban development are the most important drivers responsible for the detrimental alteration of ecosystem structure and function, which can lead to a loss of biodiversity^1,2^. Given the drastic and fast environmental changes, obtaining quantitative data and understanding the mechanisms that influence microbes and microbial communities is fundamental for predicting the impact of the microbial response to forces that drives these changes and determine their consequences, not only at local scale but at a regional and global scale^3^. Within any given ecosystem, the efficacy and intensity of microbial responses to the ecosystem largely depend on the functional identity and/or population size of the bacterial strains within the community^4,5^.

Many studies from different countries have indicated rivers water contamination not only with pesticides, metals, and pharmaceuticals but also with a spectrum of potentially allochthonous microorganisms. For example, a report from prior American study in New Jersey determined the risk of getting diseases because of the discharge of untreated domestic wastewater to the Lower Passaic River^6^. This study revealed that the concentrations of pathogens in the Passaic River surpass the recognized criteria of water quality for human consumption. Studies from India indicated that most of its rivers are heavily polluted by discharge of untreated domestic sanitary sewage, direct discharges from industrial waters, and non-point agricultural drainage^7,8^. Data from China indicate that the quality of water in most of the rivers is poor and declining owing to wastewater discharges, agricultural and aquacultural run-offs of fertilizers, pesticides, and manure, causing widespread eutrophication^9^. Several studies from the Reconquista River basin in Argentina have reported heavy bacterial contamination along the river and its tributaries^10,11^.

The Pinheiros River is located in São Paulo state, Brazil. This river links the Tiete River, the most important aquatic system of the basin, to the Billings Reservoir, which has 1,560 km^2^ of the drainage area and an estimated storage volume of 995 million m^3,12^. The severe drought and increased demand for electricity supply during the early 19th century helped spur the decision to divert the natural flow of the river into the Billing’s dam, the largest water reservoir in the state of São Paulo which supply water to more than 5.4 million people. After 1992, however, with increases in pollution and a lack of adequate wastewater treatment, the waters of the Pinheiros River were prohibited from being reversed into Billings, except in cases of flood control in São Paulo^13,14^. Pinheiros River has long suffered from anthropogenic pollution caused by nonpoint domestic sewage load that is released daily (without any treatment) on the various tributaries. Other sources of pollution, such as solid waste, are difficult to control. For example, the poorly swept streets and the contamination of soils by industrial runoff and discharges contaminate the river.

According to the Brazilian Environment National Council (CONAMA) Resolution 357/2005, which categorizes the quality of the water into five classes that range from clean to polluted, the Pinheiros River is categorized as class 4, being very polluted, which means that its water should be used only for navigation and contemplation purposes. Excessively polluted rivers like the Pinheiros strongly affect the composition of the bacterial community and this may, in turn, alter the functioning of the whole aquatic ecosystem. To date, the few articles published about this river have focused on its chemical pollution, treatment, and rehabilitation^12,13,15–19^. Information regarding the microbial communities inhabiting the river surface water are lacking.

Recently, several studies in aquatic environments have employed advanced sequencing techniques that enable access to data regarding the functional potential of a microbial community; these measurements typically focus on energy metabolism that involve the carbon, nitrogen, and sulfur cycles^20–24^.

The objectives of this study were to (i) determine the diversity and abundance of the bacterial communities in the surface water along the Pinheiros River using the 16 S rRNA gene-based Illumina MiSeq sequencing, (ii) evaluate the presence of potential pathogens in these water samples and (iii) explore the predicted functional profiles of the obtained microbial communities in the Pinheiros river to determine their role in the ecosystem.

## Materials and Methods

### Sample Collection

Water samples were collected in March of 2018 from 11 and 5 locations (between 0.1 to 2.74 km apart) of the Pinheiros River and Billings reservoir, respectively, in São Paulo. Samples from the Pinheiros were collected from bridges and obtained in the middle of the river at a depth of 10–50 cm below the water surface using a Van Dorn sampler. Five samples from the Billings reservoir were collected from the side of a boat just below the water surface (10 cm) also using a Van Dorn sampler. At each sampling event, the Van Dorn sampler was filled (8.2 liters) with enough water to fill a laboratory-sterile 250 plastic bottle. All samples were collected in duplicate. Samples were stored in a cooler (4 °C), transported to the laboratory, and stored at −80 °C before genomic DNA extraction. The temperature (Temp) and pH from each sample were determined on-site using a Multi-parameter water (YSI, USA). The water samples were also collected for analysis of dissolved oxygen (DO), turbidity, nitrate (NO3-), sulfate (SO4–2), orthophosphate (PO43−), phosphorus (P), and ammonia nitrogen (NH4+-N). The sampling site locations in the Pinheiros River and Billings reservoir in São Paulo are presented in Supplementary Fig. 1.

### DNA Isolation, Gene Amplification, and Library Preparation

Total bacterial DNA was extracted from a 10–50-mg pellet concentrated from a water sample centrifuged at 4000 g for 20 min. DNA was extracted using the PowerSoil DNA isolation kit (MO BIO Laboratories: Carlsbad, CA, USA) as per the manufacturer’s instructions. To minimize potential bias during DNA extraction, each sample was extracted as a duplicate and then pooled to quantitate DNA yield using a Qubit 2.0 fluorometer (Life Technologies: Carlsbad, CA, USA). The extracted DNA from each sample was subjected to PCR amplification of the V3-V4 variable region of the 16S rRNA gene using previously published primers Bakt_341F/Bakt_805R^25^ according to the conditions previously described by our group^26,27^. After recovery of the target bands by the Freeze N Squeeze DNA Gel Extraction Spin Columns (Bio-Rad: Hercules, CA, USA) and quantification on a Qubit 2.0 fluorometer (Life Technologies: Carlsbad, CA, USA), the amplicons from each surface group were pooled at equimolar concentration and diluted to 4 nM. Indexing of DNA and preparation of libraries were performed as previously reported^26,27^. The prepared library was finally loaded on an Illumina MiSeq cartridge for paired-end 300 sequencing.

### Bioinformatics and Statistical Analysis

Base calling and data quality were initially assessed on the MiSeq instrument using RTA v1.18.54 and MiSeq Reporter v2.6.2.3 software (Illumina Inc., CA). All 16 S rRNA sequences generated in this study were analyzed using the 16 S Microbiome Taxonomic Profiling pipeline implemented in the EzBioCloud (https://www.ezbiocloud.net/) application and the EzBioCloud Database Update 2019.04.09^28^. Sequences that could not be classified into any known group were assigned as “unclassified”. Taxonomic groups with a calculated abundance ≤ 0.3% were pooled and labeled as ETC.

For the detection of bacterial pathogens, we considered any bacteria to be potentially pathogenic if at least one species with a minimum abundance of 10 strains of any genus was categorized as biosafety level 2 or 3 by the American Biological Safety Association (https://my.absa.org/tiki-index.php?page=Riskgroups).

### Taxonomic and functional biomarkers

Biomarker analysis was conducted using LEfSe to determine the significant differences in microbial abundance between the sequencing data of Pinheiros River and the unpublished data from a similar experiment performed during the same sampling period from five untreated surface water samples collected from the Billings reservoir in Sao Paulo. Billings reservoir was chosen for this analysis because the water of the Pinheiros River may somehow mix with the Billings reservoir at a certain circumstance. For instance, the pumping of the Pinheiros River to the Billings reservoir is allowed by Brazilian regulations in cases of flood control, a need for emergency power generation, and other exceptional situations^13^. Linear discriminant analysis (LDA) of effect size (LEfSe) was used to identify biologically and statistically significant changes in the relative OTU relative abundance of microbial taxa^29^. Functional predictions were generated using PICRUSt with reference to the Kyoto Encyclopedia of Genes and Genomes (KEGG) Ortholog^30^ using the taxonomy generated from the EzBioCloud Database Update 2019.04.09.

### Sequence data availability

All sequence data described here are available in the online Zenodo repository: 10.5281/zenodo.3380549.

## Results

### Physicochemical characteristics of water samples

Physical and chemical characteristics are summarized in Table 1. The colors of all samples were dark green with gas bubbling and completely absent of aquatic life due to extremely depleted dissolved oxygen “DO”. The depletion of DO is likely due to its large volume consumption by the microbial activity and organic pollutants. Also, the gross contamination of the river contributes to high turbidity of water and an intense (foul) smell of rotten egg. On average, the surface water temperature was 23.6 °C, which accelerated the growth of phytoplankton bloom. All the samples had pH values in the range of 5.5 to 6.5. Total nitrogen ranged from 9 to 25.7 mg/L and nitrate-nitrogen ranged from 2.1 to 7.1 mg/L. The highest total phosphorus was measured in sample P12 and the lowest in sample P02.

**Table 1:** Physio-chemical characteristics of water samples from the Pinheiros River.

Sample ID	DO* mg/l	Temp.	pH	Sulfate mg/L	Orthophosphate mg/L	Phosphorus mg/L	Ammonia mg/L	Nitrate mg/L
P1	1.2	24.1	6.5	0.4	0.24	0.08	0.9	6.9
P2	2.3	25	6.3	0.5	0.66	0.22	16.1	2.8
P3	1.4	24.3	6.17	0.7	0.68	0.23	17.9	5.5
P5	3.9	22.8	5.84	2.9	1.49	0.49	24.4	2.1
P6	0.5	23.1	6.06	0.6	1.59	0.53	22.7	3.2
P7	3	23.1	6.2	0.5	1.68	0.56	23.9	3.8
P8	1.2	24.2	6.2	0.6	1.61	0.54	21.8	2.7
P9	1.3	23.2	6.25	0.4	1.71	0.57	22.8	3.8
P10	0.8	24.1	6.27	0.6	1.66	0.55	24.1	7.1
P11	3.2	23	6.09	0.6	1.38	0.46	20.2	6.5
P12	2.1	22.6	5.9	0.5	1.77	0.59	25.7	4.6

*Dissolved Oxygen.

### The Microbial Community Diversities of the Pinheiros River

The total number of filtered reads generated from the eleven libraries on the Illumina MiSeq platform was 2,712,455 (Min: 111,825 in P1; Max: 364,534 in P7), presented in Table 2. To minimize computational time, 100,000 reads from each sample were automatically selected, cleaned, and analyzed by the EzBioCloud. This resulted in a total of 731,089 (Min: 61,590 in P3; Max: 76,152 in P12) valid reads after quality filtering with an average of 99.1% coverage of library sequences with a mean length of 453 bp. The higher value of coverage indicates a considerably high number of libraries in each sample and reflects the actual species’ population detected in each sample. All those sequences were clustered into OTUs identified at the species level and ranged from 2.685, detected in P7, to 3.747 in P1. The distribution of sequence lengths produced agreed with the 464 bp amplicon length of the 16 S rRNA. The indices of alpha diversity of the 11 surface water samples were computed at cut-off levels of 3% using the diversity indices of bacterial richness (ACE, Chao1, and Jackknife) and evenness (Shannon index, Simpson function, and NPShannon). The sequencing depth and coverage (as demonstrated in Table 2), and the rarefaction analysis shown in Supplementary Fig. 2 all indicate that deep sequencing was successfully performed in this study. Moreover, phylogenetic diversity was also used to measure biodiversity by incorporating the phylogenetic difference between species. As expected, the overall results revealed no remarkable difference in the bacterial species composition and abundances or the richness, evenness, and heterogeneity between the sampling sites. The relationships between the collected samples were investigated using UniFrac based principal coordinate analysis (PCoA). This analysis revealed that clustering of samples was according to the grouping of the 16S rRNA dendrogram rather than samples as depicted in Supplementary Fig. 3.

**Table 2:** The number of raw and valid reads sequenced for each sample, number of species and OTUs found, and subsequent alpha diversity measures.

Sample ID	Raw reads	Valid reads	No. of reads identified at the species level	No. of species found	No. of OTUs found in the sample	Good’s coverage of library(%)	Alpha diversity indices		*Jackknife*	*Shannon*	*Simpson*	*NPShannon*	*Phylogenetic diversity*
							*ACE*	*Chao1*					
P1	111,825	66,731 (66.7%)	40,356 (60.5%)	1,753	3747	99.1	4,142	#####	4,373.00	5.713	0.026	5.803	3,663.00
P2	113,528	68,110 (68.1%)	41,004 (60.2%)	1,693	3302	99	#####	#####	3,958.00	5.286	0.038	5.366	3,469.00
P3	137,636	61,590 (61.6%)	37,474 (60.8%)	1,518	3461	99.1	#####	#####	4,044.00	5.669	0.024	5.76	3,102.00
P5	361,826	64,056 (64.1%)	50,269 (78.5%)	1,730	2978	99	3,463	#####	3,638.00	5.251	0.046	5.325	3,245.00
P6	187,729	70,130 (70.1%)	57,596 (82.1%)	1,740	2924	99.2	3,333	#####	3,502.00	5.229	0.048	5.298	3,253.00
P7	364,534	65,799 (65.8%)	52,920 (80.4%)	1,591	2685	99.1	#####	#####	3,283.00	5.139	0.028	5.208	3,031.00
P8	181,507	62,000 (62.0%)	43,977 (70.9%)	1,528	2983	99.1	#####	#####	3,517.00	5.495	0.023	5.573	2,995.00
P9	226,645	69,846 (69.8%)	58,839 (84.2%)	1,687	2761	99.2	#####	#####	3,353.00	4.914	0.073	4.981	3,196.00
P10	330,664	64,563 (64.6%)	51,004 (79.0%)	1,663	2940	99.1	#####	#####	3,551.00	5.185	0.046	5.262	3,145.00
P11	339,109	62,112 (62.1%)	48,849 (78.6%)	1,552	2690	99.1	3,085	#####	3,249.00	5.018	0.055	5.092	3,009.00
P12	357,452	76,152 (76.2%)	63,547 (83.4%)	1,761	2928	99.2	#####	#####	3,559.00	5.041	0.064	5.105	3,211.00

### The Microbial Community Structure in the Pinheiros River

**Figure 1:**
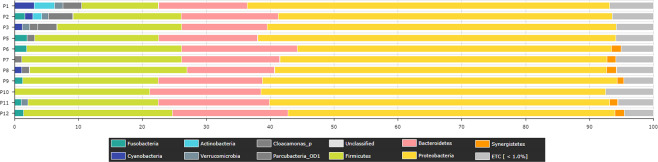
Phylum level taxonomical abundance ratio from each surface water sample in the Pinheiros River. Only bacterial phyla that had a relative abundance of 1% or greater are presented.

The majority of the identified OTUs reads from the eleven surface water samples, defined by 97% sequence similarity, were affiliated with 19 known phyla and ETC with a median abundance value of 1.89% (Min: 1.52% in P9; Max: 2.23 in P3). The most abundant phylum was *Proteobacteria* (median abundance value 53.4%, min: 49.1% in P6; max: 56.4% in P1) followed by *Firmicutes* (median abundance value 21.1%, min: 12% in P1; max: 25% in P7), and *Bacteroidetes* (median abundance value 15.6%, min: 13.3% in P3; max: 18.3% in P6) (Fig. 1 & Supplementary Table S1**)**. Among the *Proteobacteria*, the *ε-Proteobacteria* (median abundance value 22%, min: 8.1% in P7; max: 28.6% in P9) and *β-Proteobacteria* (15.3%) were the most dominant classes in all samples, followed by *δ-Proteobacteria* (10.2%, min: 6.5% in P9; max: 22.7% in P2). The predominant *Firmicutes* belonged to the class *Clostridia*. The order *Clostridiales* (median abundance value 13.3%, min: 7.8% in P1; max: 15.6% in P8) was mainly represented by the *Ruminococcaceae* (5.4%), *Lachnospiraceae* (2.4%), *Mogibacterium_f* (1.9%), and *Flavobacteriaceae* (3.29%) families. The predominant *Bacteroidetes* belonged primarily to the classes *Bacteroidia* (order *Bacteroidiales*, family *Porphyromonadaceae*, *Prevotellaceae*, *Bacteroidaceae*, *AC160630_f*, *Lentimicrobiaceae*, and *Prolixibacteraceae*) and *Flavobacteria* (order *Flavobacteriales*, family *Flavobacteriaceae*). *Actinobacteria*, *Tenericutes*, *Chloroflexi*, *Fusobacteria*, *Synergistetes*, *Verrucomicrobia*, *Cyanobacteria*, and *Spirochaetes* were profiled as rare phyla with median abundances of 0.51, 0.17, 0.15, 1.1, 0.95, 0.89, and 089%, respectively.

**Figure 2:**
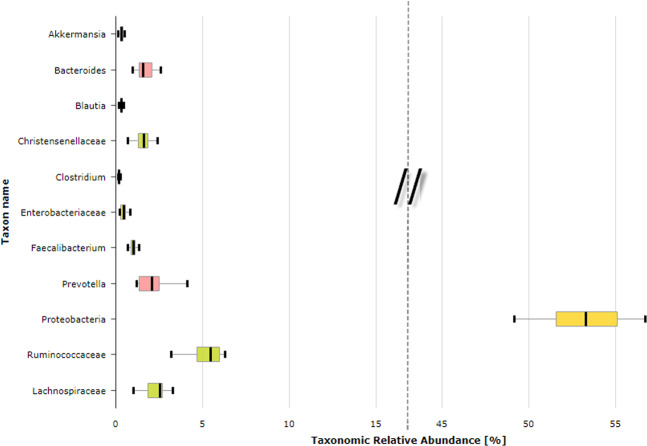
Average composition of important human gut bacteria detected in the water surface of the Pinheiros River.

**Figure 3:**
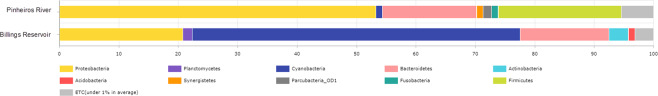
Comparison of the averaged taxonomic compositions of the bacteriome in the Pinheiros River and Billing Reservoir. Only bacterial phyla that had a relative abundance of 1% or greater are presented.

The search for predefined bacterial groups in the Pinheiros River revealed important taxa associated with the human gut that included the phylum *Proteobacteria* (median abundance value 53.3%, min: 49.2%, max: 56.7%) and the families *Ruminococcaceae* (median abundance value 5.5%, min: 3.2%, max: 6.3%), *Lachnospiraceae* (median abundance value 2.5%, min: 1.0%, max: 3.3%), and *Christensenellaceae* (median abundance value 1.6%, min: 0.7%, max: 2.4%) (Figs. 2 and 3). Unsurprisingly, these results indicate the heavy polluted Pinheiros River suffers from anthropogenic pollution.

### Compositional Differences in Bacterial Diversity between the Pinheiros River and Billings Reservoir

As expected, significant compositional differences in bacterial microbiota were found between the Pinheiros River and Billings’s reservoir (p < 0.001). The relative abundance of *Proteobacteria* (53%) was higher in Pinheiros samples than in Billings samples. *Cyanobacterium* (55%) was more abundant in Billings water (Supplementary Fig. 3) than in the Pinheiros River, which had only 1%. Moreover, *Firmicutes* (21%) was the second most abundant phylum in the Pinheiros River and it was undetectable in Billings reservoir. All diversity measures (richness, ACE, Chao1, and Shannon index) were found to be significantly higher (p < 0.05) in the Pinheiros River compared to the Billings reservoir. The PCoA analysis also revealed significant differences (p < 0.05) between both aquatic ecosystems (data not shown).

**Figure 4:**
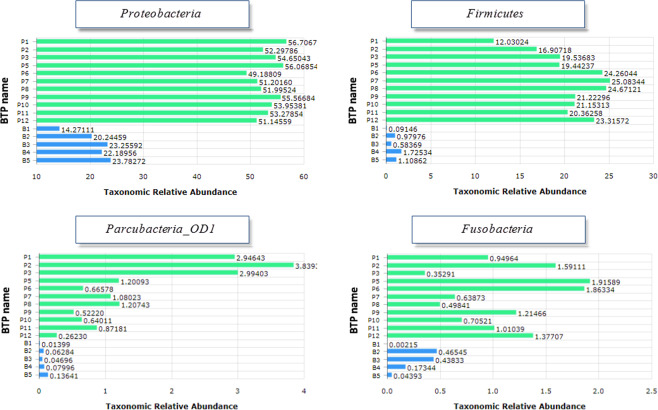
LEfSe analysis between the Pinheiros River and Billings Reservoir. Histogram of the LDA scores computed for most OTUs are differentially abundant across groups.

Next, we applied the LEfSe analysis to identify significant taxonomic biomarker differences between the Pinheiros River and Billings reservoir. The results revealed differential species of 23 phyla, 51 classes, 96 orders, and 176 families at a false discovery rate (FDR) ≤ 0.05 and logarithmic LDA scores ≥ 2.0 (Supplementary Table S2). *Proteobacteria* (including the class *Epsilonproteobacteria*), *Firmicutes* (including the class *Clostridia*), *Parcubacteria_OD1* (including the class LCGL), and *Fusobacteria* (including the class *Fusobacteria_c*) were the most abundant in the Pinheiros River (Fig. 4).

### Functional Predictions

Functional predictions generated by PICRUSt and LEfSe yielded 20 statistically significant (p (FDR) < 0.05) enriched KEGG categories between Pinheiros River and Billings reservoir microbiome in the relative abundance of microbial genes related to metabolic pathways. Compared to the Billings reservoir, 12 KEGG pathways in the Pinheiros River exhibited higher abundance of genes that code for pathways involved in cellular processes (bacterial chemotaxis and flagellar assembly), metabolism (carbon metabolism, biosynthesis of antibiotics, carbon fixation pathways in prokaryotes, biosynthesis of secondary metabolites, biosynthesis of amino acids and microbial metabolism in diverse environments), environmental information processing (ABC transporters, two-component system, and bacterial secretion system), and the genetic information processing pathway (ribosome). The remaining 8 pathways related to human disease (tuberculosis and human papillomavirus infection), metabolism (arginine and proline metabolism, photosynthesis, steroid biosynthesis, and photosynthesis - antenna proteins) environmental information processing (cell adhesion molecules (CAMs)) and organismal systems (parathyroid hormone synthesis, secretion and action) were significantly enriched in the Billing’s reservoir (Supplementary Table S3).

Based on the criteria of pathogen identification described in the Materials and Methods, 35 potential pathogenic bacterial genera were identified. Among the 11 samples investigated, the genus *Arcobacter* was the predominant potentially pathogenic genus, with a median relative abundance of 21.6% and range of 7.8–28.1%. The analysis also indicated that 12 of the 35 potential pathogenic genera displayed abundance ratios of >0.1% in at least one of the 11 samples tested (Supplementary Table S4).

## Discussion

In this study, we employed the 16S rRNA gene Illumina MiSeq sequencing for the first time to profile the structure of the bacterial community and diversity of 11 surface water samples collected from the Pinheiros River in the city of São Paulo. *Proteobacteria* and *Firmicutes* were the dominant phyla, which together represented > 70% of all sequences obtained. The third most abundant phylum was found to be *Bacteroidetes*. The *Proteobacteria*, *Firmicutes*, and *Bacteroidetes* have been detected in domestic sewage sludges from São Paulo, Brazil^31^ and China^32,33^. The dominance of *Proteobacteria* and *Bacteroidetes* has also been found in some freshwater environments^34,35^ while those of the lake sediment samples were dominated by sequences affiliated with *Firmicutes*^36,37^. Frequent detection of these three phyla have also been reported in microbial fuel cells^38^ that generate electricity through the oxidation of organic matter under anaerobic conditions^39^. *Proteobacteria* are the largest phylum within the bacteria domain and contain a very high level of bacterial metabolic diversity related to global carbon, nitrogen and sulfur cycling. The previous study by Takai and colleagues demonstrated that members of *Proteobacteria*, particularly *Epsilonproteobacteria*, possess the ability to conduct energy metabolism using reduced sulfur compounds and carbon assimilation via the reductive tricarboxylic acid cycle^40^. Thus, the 53.3% overrepresentation of *Proteobacteria* detected in this study might be connected with the reduction of DO levels in the Pinheiros River. Besides the *Proteobacteria*, the high loads of organic carbon coupled to poor nutrition, and hence, the subsequent oxygen depletion in the Pinheiros River favor growth of other bacterial community members that are active under anoxic and sub-oxic conditions, such as the bacterial fermenters *Clostridiales* (median abundance 13.3%) and the denitrifying members of *Rhodocyclales* (median abundance 12%). Bacterial representatives of the *Proteobacteria* were well-represented in all 11 samples, with *Arcobacter cryaerophilus* as the dominant species. This bacterial species, together with other strains of *Arcobacter* have been previously isolated from different types of humans, animals, foods, and areas of the environment^41–45^ and are considered emergent enteropathogens and potential zoonotic agents^43,46,47^. A previous study by Collado and colleagues^41^ revealed a strong correlation between the concentration of bacterial indicators of human fecal signature in water and the detection of *Arcobacter spp*. The same study concluded that the persistence of these bacteria in wastewater indicates that this could be one ecological reservoir. Thus, it is not surprising that the higher abundance of *Arcobacter cryaerophilus* in the Pinheiros River may indicate heavy fecal contamination. Nevertheless, our results replicate the findings of the recent metagenomic sequencing studies that revealed a higher prevalence of *Arcobacter* in sewage ecosystems and wastewater treatment plants^48–50^ and provides further support to the suggestion that *Arcobacter* is primarily planktonic^51^. In one study, a metagenomic assembly of the near-complete genome of *Arcobacter cryaerophilus* from untreated sewage influent samples recovered 25 putative antibiotic resistance genes^52^. Jacquiod *et al*.^53^ described *Arcobacter* from wastewater treatment plants as a keystone player involved in shuttling antibiotic resistance genes between distant gram‐positive and gram‐negative phyla. It has also been reported that the *Arcobacter spp* are associated with conjugative plasmid transfer^54^ and survival in changing environmental conditions^55^. The second most abundant bacterial species detected in the Pinheiros River is the well-characterized exoelectrogenic *Geobacter_uc* from the *δ-Proteobacteria* class^56^. High concentrations of *Geobacter* species are often observed in subsurface environments when dissimilatory metal reduction is an important process particularly in environments that have been subject to anthropogenic influences^57^. The previous molecular study by Holmes *et al*.^58^ reported high enrichment of *Geobacteraceae* during the reduction of the soluble oxidized form of uranium U(VI) in a variety of sediments samples. This metal reducer bacterial group has also been found to reduce elemental sulfur to sulfide^59^ and can also reduce the number of other inorganic electron acceptors, including nitrate and U^6+^, ^60^. Thus, the abundance of *Geobacter* species in this study may partly explain the high concentration of sulfide inorganic anion in the Pinheiros River. Consequently, the sulfide compounds combine with hydrogen to produce hydrogen sulfide gas that is responsible for the annoying and distinctive rotten-egg odor associated with the Pinheiros River. The genomes of the *Geobacter species* are known to have multiple copies of chemotaxis genes that play a vital role in sustaining cell growth and survival in a variety of environmental conditions.

Various physicochemical water parameters, such as temperature, DO, ammonia, Phosphorus, and Orthophosphate concentrations are reported to influence the dynamics of the bacterial populations in aquatic ecosystems^61,62^. In this study, all 9 physicochemical properties investigated were significantly altered and were thus considered to be potential key regulators of the bacterial distribution, abundance, structure or potential activity in the eutrophic Pinheiros river. It is conceivable that the high concentrations of nutrients, such as orthophosphate, ammonia, and phosphorus are critical for enriching genes involved in bacterial chemotaxis and flagellar assembly, which has been reported in other aquatic ecosystems. Therefore, it is not surprising that the bacterial species in the water environment are capable of motility^63^ and that their motion is controlled by chemotaxis. Consequently, it appears that certain factors in the Pinheiros River contribute to the selection of bacterial functions as well as their clustering, and that their byproducts effectively permit their adaptation by increasing their response to new selective pressures. As a result, this provides an ecological rationale for the broad diversity of the bacterial population detected in the Pinheiros River.

The main limitation of this study is that it was restricted to a single sample at a single point in time. However, we believe that the heavy pollution of the Pinheiros River and its poor quality can be adequately described by a single sample. Also, we used bacterial DNA genomics for this investigation, which would have revealed the presence of bacterial populations regardless of whether they are dead or alive, culturable cells, or non-culturable cells. While the transcriptomic-based strategy is considered a superior approach for the proper estimation of the living bacterial community and for illuminating the activity of microbial functional genes^64^, it should be noted that manipulating RNA is more difficult than DNA in terms of stability and extraction^65^.

## Conclusions

Our study is the first of its kind to assess and provide a comprehensive assessment of the diversity of the bacterial community and functions in the Pinheiros River. The data presented here agree with the expected bacterial populations thriving in sewage-contaminated environments. Certain environmental variables such as phosphate, ammonia-nitrate, and DO were found to be important factors that structured bacterial communities within this ecosystem. Analysis of the potential functions of the bacteriome indicated that the River had higher relative abundances of genes encoding for bacterial chemotaxis and flagellar assembly. These results enhance our knowledge regarding the bacterial composition in the Pinheiros river.

## Supplementary Information

Supplementary Information.

Supplementary Table 1.

Supplementary Table 2.

Supplementary Table 3.

Supplementary Table 4.
