# Supplementary material for: Bacterial community composition and potential pathogens along the Pinheiros River in the southeast of Brazil
Source: Sci Rep. 2020 Jun 9;10:9331. doi: 10.1038/s41598-020-66386-y (PMC7283273; doi:10.1038/s41598-020-66386-y)
Supplement: Supplementary file 1 — Supplementary Information. [file 41598_2020_66386_MOESM1_ESM.docx]

Supplementary Information for:

**Bacterial community composition and potential pathogens along the Pinheiros River in the southeast of Brazil**

Rafaela Garrido Godoy^1$^, Marta Angela Marcondes^2$^, Rodrigo Pessôa^1^, Andrezza Nascimento^1^, Jefferson Russo Victor^1, 3^, Alberto José da Silva Duarte^1, 4^, Patricia Bianca Clissa^5^, Sabri Saeed Sanabani^6^*

^1^ Laboratory of Dermatology and Immunodeficiency*,* São Paulo University Medical School, Sao Paulo, SP, Brazil.

^2^ Department of Microbiology, Fedral University of São Caetano, São Paulo, Brazil.

^3^Division of Environmental Health, Faculdades Metropolitanas Unidas (FMU), Laureate International Universities, São Paulo, Brazil.

^4^Division of Pathology, Medical School, University of São Paulo, São Paulo, Brazil.

^5^Immunopathology Laboratory, Butantan Institute, Sao Paulo-SP, Brazil

^6^Laboratory of Medical Investigation LIM 03, Hospital das Clínicas (HCFMU), School of Medicine, University of São Paulo, São Paulo, Brazil.

* Corresponding author

Sabri Saeed Sanabani, PhD

E-mail: sabyem_63@yahoo.com

Laboratory of Dermatology and Immunodeficiency, LIM56/03.

Instituto de Medicina Tropical de São Paulo

Faculdade de Medicina da Universidade de São Paulo

Av. Dr. Eneas de Carvalho Aguiar, 470 3º andar

São Paulo Brazil 05403 000

Phone: + 5511 3061 7194 ext:218


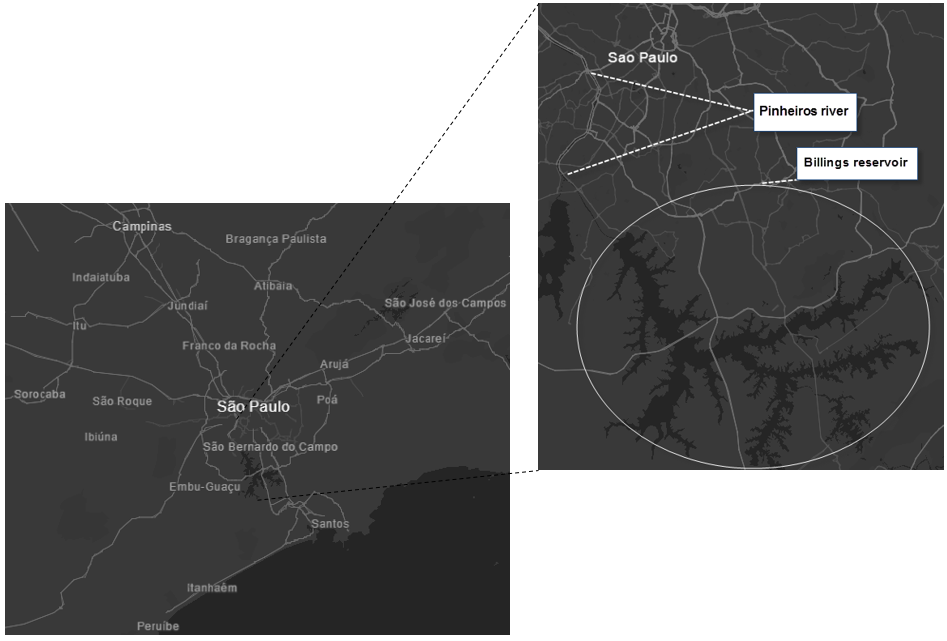


**Supplementary Fig 1**: Map showing sampling site locations in the Pinheiros River and Billings reservoir in São Paulo. Map was obtained from HERE XYZ map studio <https://www.here.com/products/mapping/studio>.


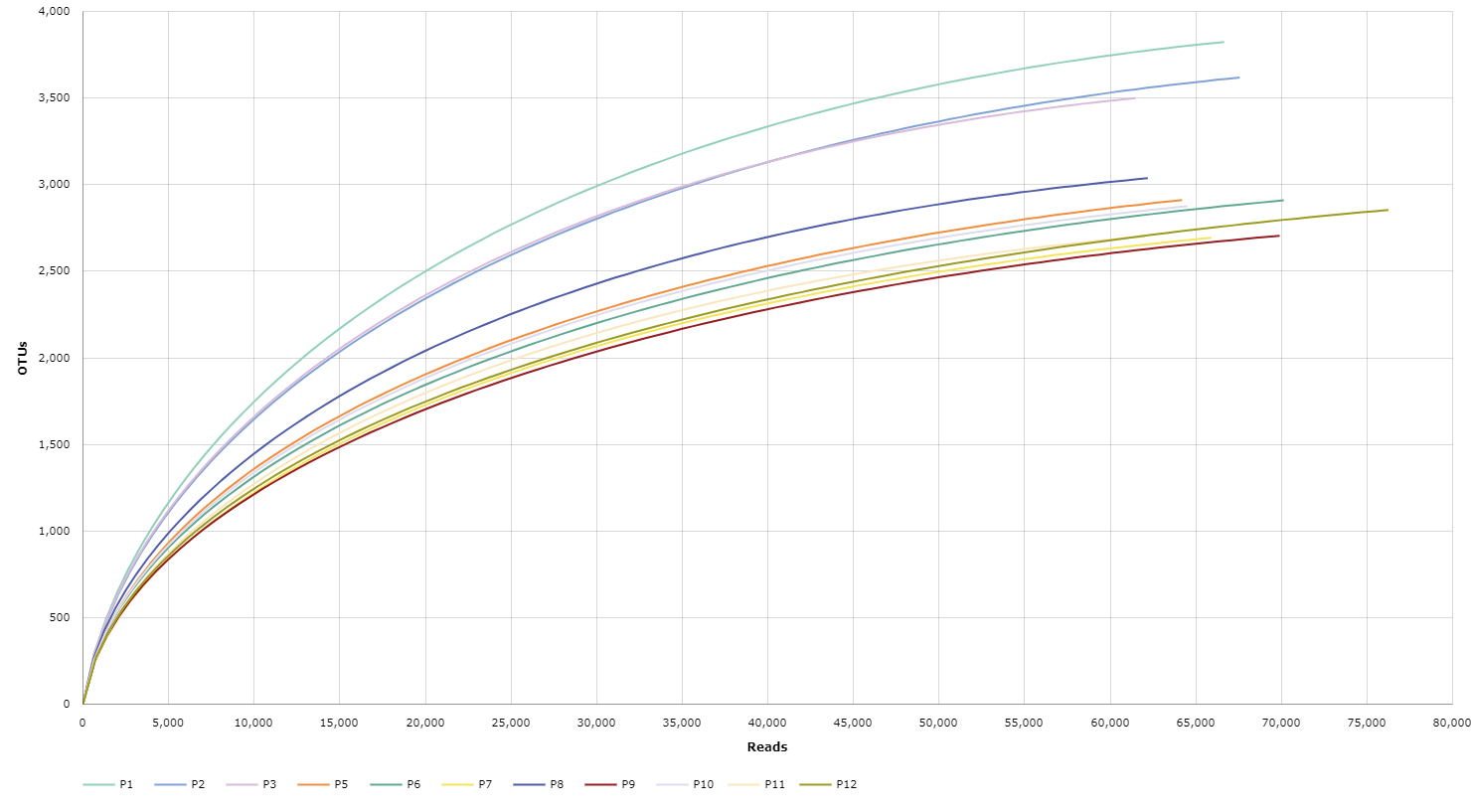


**Supplementary Fig 2:** Rarefaction curves of OTUs (operational taxonomic units) clustered at 97% sequence identity across the 11 water surface samples.


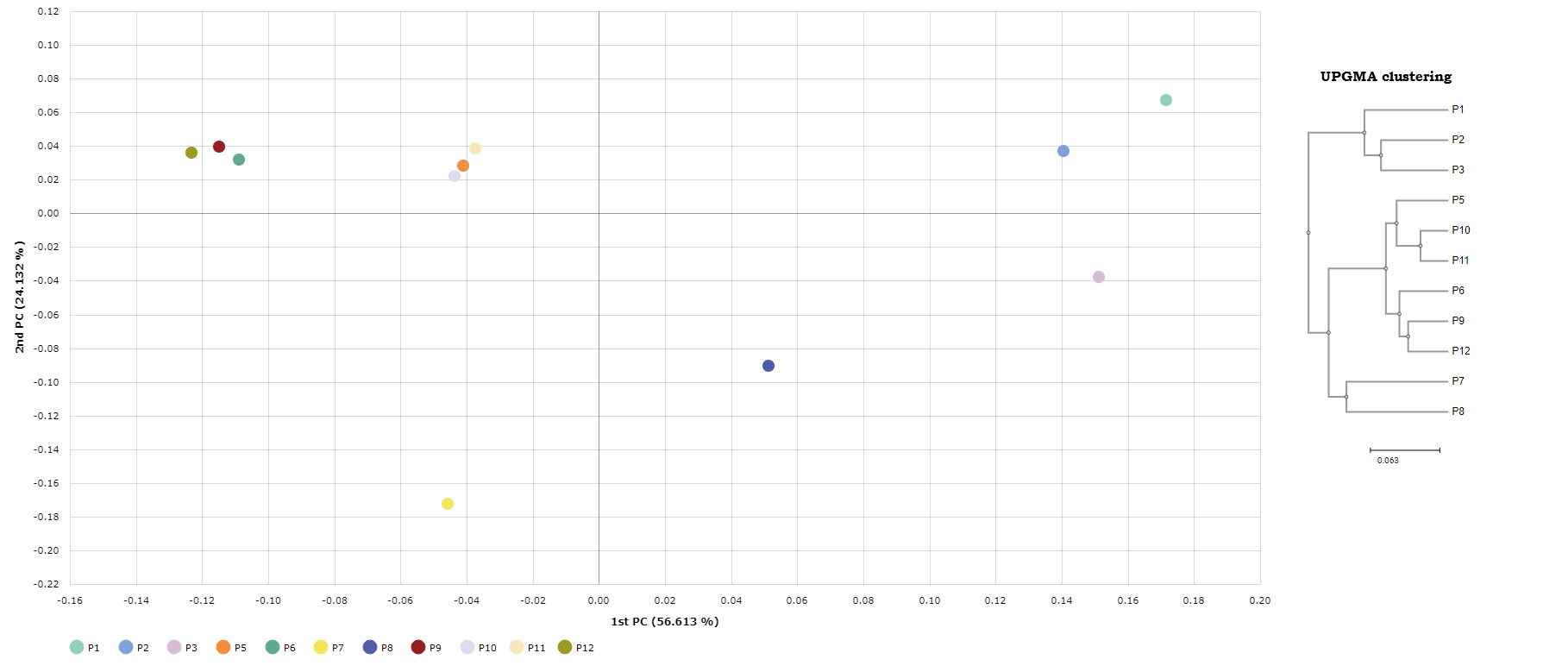


**Supplementary Fig 3**: Principal components analysis and UPGMA clustering of beta diversity. Similarities based on diversity across samples were investigated using PCoA and UPGMA clustering after rarefaction, and explained 80.74% of the variance. Beta diversity was computed using weighted Unifrac distances.
